# Fabrication of a High‐Quality, Porous, Surface‐Confined Covalent Organic Framework on a Reactive Metal Surface

**DOI:** 10.1002/cphc.201600027

**Published:** 2016-02-05

**Authors:** Christian R. Larrea, Christopher J. Baddeley

**Affiliations:** ^1^ EaStCHEM School of ChemistryUniversity of St. AndrewsSt. AndrewsFifeKY16 9STU.K.

**Keywords:** alloys, nanostructures, scanning probe microscopy, surface chemistry, thin films

## Abstract

A major goal of heterogeneous catalysis is to optimize catalytic selectivity. Selectivity is often limited by the fact that most heterogeneous catalysts possess sites with a range of reactivities, resulting in the formation of unwanted by‐products. The construction of surface‐confined covalent organic frameworks (sCOFs) on catalytically active surfaces is a desirable strategy, as pores can be tailored to operate as catalytic nanoreactors. Direct modification of reactive surfaces is impractical, because the strong molecule–surface interaction precludes monomer diffusion and formation of extended architectures. Herein, we describe a protocol for the formation of a high‐quality sCOF on a Pd‐rich surface by first fabricating a porous sCOF through Ullmann coupling on a Au‐rich bimetallic surface on Pd(111). Once the sCOF has formed, thermal processing induces a Pd‐rich surface while preserving the integrity of the sCOF architecture, as evidenced by scanning tunneling microscopy and titration of Pd sites through CO adsorption.

Increasingly, selectivity is a major consideration in industrial‐scale catalysis. Heterogeneous catalysts consisting of metal particles dispersed on a support material with a high surface area often possess a multitude of active catalytic sites. The lack of control over the structure of such sites is often detrimental to selectivity. A successful strategy in enantioselective heterogeneous catalysis is to modify a metal surface through the adsorption of chiral molecules, which provide chiral active sites for catalytic reactions. A limitation of this approach is the optimization of the surface coverage of modifiers and the instability of the modified surface under reaction conditions.^1^ A strategy to introduce selectivity may come from the formation of robust porous covalent architectures that are able to host guest molecules. Such architectures have been successfully constructed from molecular precursors on inert surfaces.^2^ However, on the more catalytically active metals, the dominant molecule–surface interaction results in molecular precursors being required to overcome high diffusion energy barriers in order to assemble.^3^ Hence, thermally activated decomposition is likely to overwhelm the formation of well‐defined surface architectures. The first major challenge is the integration of the two concepts: controlled surface modification while maintaining surface reactivity. This paper describes the construction of surface‐confined covalent organic framework (sCOF) scaffolding on a reactive metal surface. Subsequent chemical tailoring of the pores may allow for the introduction of suitable functionalities that can direct the selectivity of a reaction via specific reagent–pore interaction (Scheme 1).

**Scheme 1:**
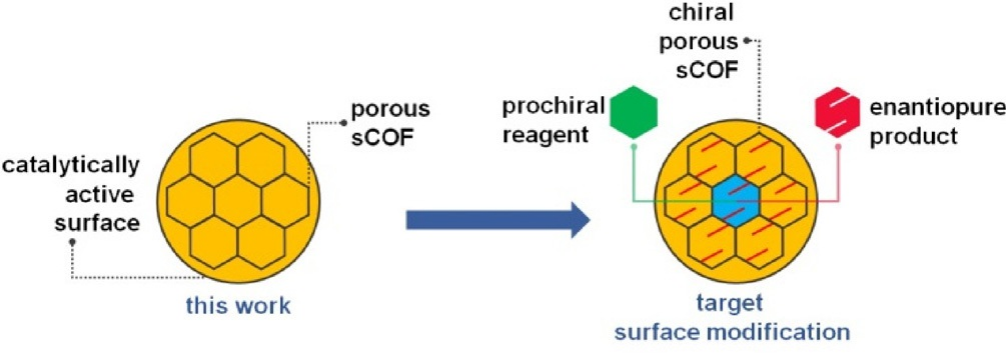
Construction of a sCOF scaffold on a catalytically active surface described in this work (left), targeting application in enantioselective surface through pore functionalization (right).

Scanning tunneling microscopy (STM) was used to investigate the deposition of the molecular precursor 1,3,5 *tris*(4‐bromophenyl) benzene (TBPB) on Pd(111) in ultrahigh vacuum (UHV), which results in dissociative adsorption with cleavage of the C−Br bond readily occurring at 300 K (Scheme 2). The Y‐shaped features associated with the activated precursor are clearly resolved, along with smaller circular features that tend to pack in hexagonal arrays (Figure 1 a). The circular features are separated by 5.6±0.7 Å, and are assigned to Br adatoms derived from C−Br cleavage, which is to be expected at 300 K given the relatively high activity of Pd.^4^

**Scheme 2:**
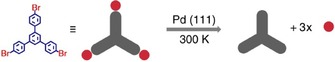
1,3,5 *Tris*(4‐bromophenyl) benzene (TBPB) and schematic of the debromination reaction on Pd(111) at 300 K. The C−Br bond is cleaved, leaving an on‐surface‐stabilized radical and co‐adsorbed Br adatoms.

**Figure 1:**
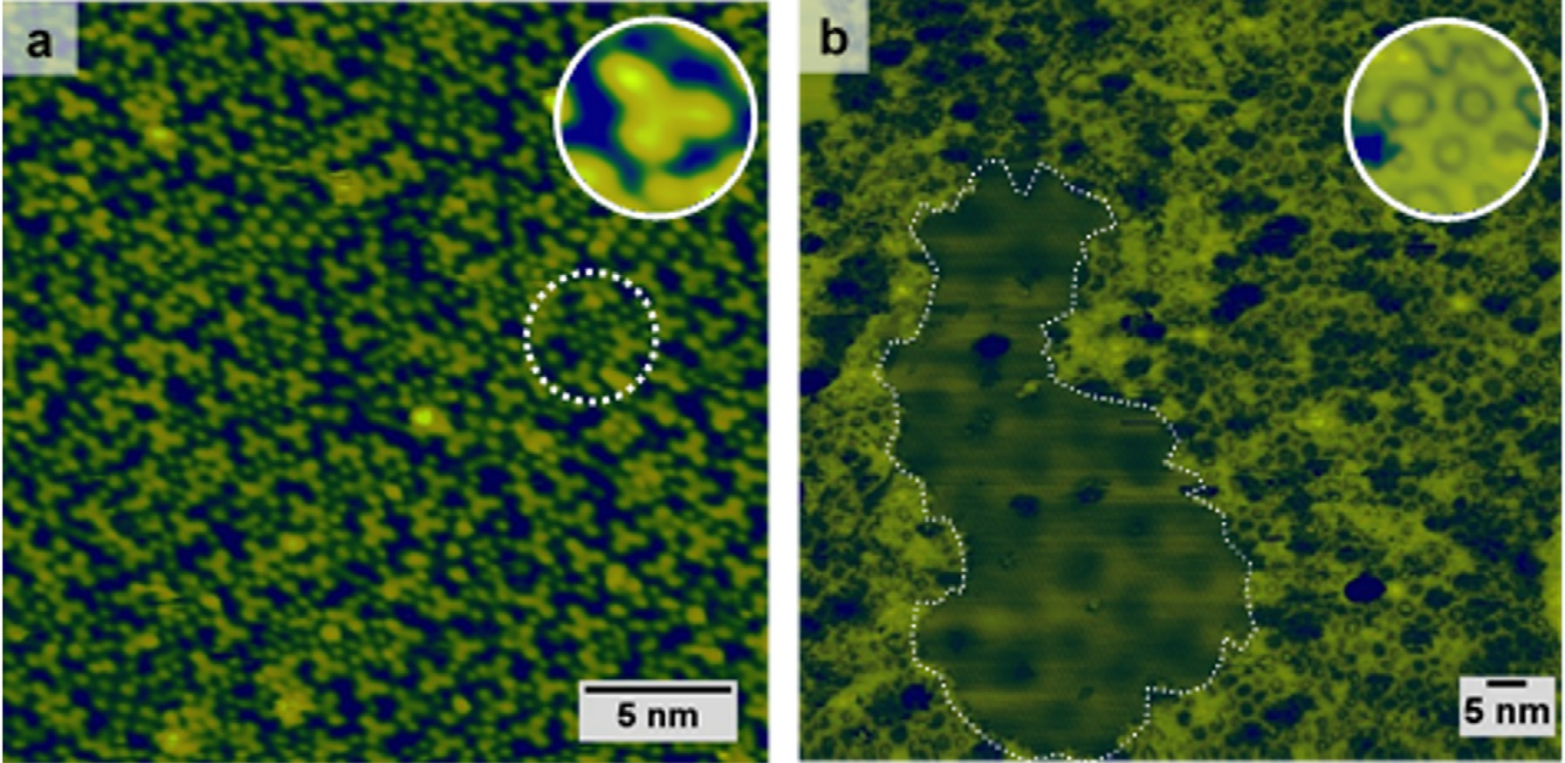
STM images of a) TBPB deposited on Pd(111) at 300 K; the inset shows a close‐up of a feature corresponding to a debrominated TBPB molecule. The dashed circle highlights hexagonally arranged split‐off Br adatoms. b) Porous sCOF formed upon deposition of TBPB on the Au–Pd(111) held at 475 K; the inset shows a close‐up of filled pores. The dashed perimeter indicates the boundary of a Br island within the sCOF on top of a Moiré pattern (−0.6 V, 300 pA).

Annealing to (or deposition at) 475 K induces extensive decomposition of the monomer, with no evidence of self‐assembly or formation of covalent structures (see Figure S1 in the Supporting Information). Similarly, Morchutt et al. deposited TBPB on a Ni(111) surface with comparable results, but they were only able to observe C−C coupling after electronic decoupling of the surface by growing a monolayer of graphene.^5^ It can be deduced from this behavior that the diffusion energy barrier of the activated precursor on Pd(111) must be sufficiently large, as it cannot be overcome even by annealing at this temperature.

Blunt et al. studied the adsorption of TBPB on Au(111) under UHV, and demonstrated that the deposition of TBPB with the sample held at 410 K facilitates the diffusion of the activated precursors, resulting in a porous sCOF that could be extended over the entire Au surface.^2^ We anticipated that the deposition of TBPB on a gold‐rich Au–Pd(111) surface alloy under similar conditions should closely emulate the surface chemistry on Au(111), and that a porous sCOF would form. A valuable feature of the AuPd system is that a solid solution is formed over the whole composition range, allowing all Au/Pd ratios to be accessed by simple annealing. As we found that the network presents remarkable thermal stability on Au(111), we conjectured that palladium enrichment of the surface could be prompted by thermal annealing whilst preserving the integrity of the sCOF. The deposition of four monolayer equivalents (MLE) of Au on Pd(111) resulted in a long‐range hexagonal Moiré pattern with an apparent modulation maxima repeat distance of approximately 7.5 nm (see Figure S2 in the Supporting Information). The Moiré pattern is a consequence of the 4.9 % mismatch in the lattice parameters of Au and Pd and provides evidence that the composition of the surface layer is almost purely Au (at least strongly Au‐rich) under these preparation conditions. We found that the deposition of TBPB on the Au–Pd(111) surface alloy held at 475 K produces an aperiodic porous network (Figure 1 b). The apparent length of a dimer within the network (13.5±0.8 Å), as measured from the centroid of the middle phenyl ring, is in reasonable agreement with two C−C coupled monomers.^2^ Metal adatom incorporation has often been reported for metastable protopolymers on surfaces, giving longer intermolecular spacings and protrusions in STM images of molecule–adatom–molecule junctions. No evidence was found for metal adatom incorporation in the present study.^6^

By analyzing the topography of one STM image (40×40 nm^2^), a pore‐size distribution reveals that the sCOF is comprised of about 45 % hexagonal (*A*=3.0±0.4 nm^2^) and 40 % pentagonal (*A*=2.0±0.5 nm^2^) pores, whereas heptagonal (12 %) or rarely square pores (3 %) account for the remainder. The percentage of hexagonal pores is close to that found on Au(111) by Blunt et al.^2^ (50 %) and we can, therefore, conclude that the morphology of the sCOF on the alloy is very similar to the one obtained on the Au(111) surface.

Along with the framework, islands of periodically arranged close‐packed protrusions on top of a Moiré background were imaged. We interpret these features as bromine adatoms, which are the by‐product of the on‐surface reaction. The apparent Br–Br interspacing distance is 6.8±0.1 Å, and the superstructure is rotated approximately 20° from the direction of the Moiré pattern. These dimensions are consistent with a commensurate (√7×√7)*R* 19.1° superlattice (see Figure S3 in the Supporting Information). This superstructure has previously been observed as a phase transition from the Au(111)–(√3×√3)*R* 30°–Br.^7^ The periodicity of the Moiré pattern is the same as that observed for the as‐prepared surface alloy and does not arise because of the lattice mismatch between Br adatoms and the surface.

STM revealed that most of the pores appeared to host adsorbed species (Figure 1 b, inset). Although we observed monomers and dimers trapped in closed and open pores, these were clearly imaged as Y‐shaped features. Instead, we believe that Br adatoms populate the pores in the framework. The pore dimensions are large enough to host at least four Br adatoms (for a hexagonal pore and assuming the same packing as in the island). However, it was not possible to image individual atoms by contrast to the atomically resolved Br island within the same image. This could either be a consequence of Br diffusion inside the pore occurring faster than the STM imaging time or because of electronic effects such as the confinement of surface states by the pores, which can differ considerably from the clean surface and, in turn, affect the imaging.^8^

To assess the thermal stability of the sCOF, the sample was annealed to progressively higher temperatures for 10 min before the images were acquired at 300 K. Some pores remained populated even after annealing at 675 K (Figure 2 a, inset), and Br desorbs completely from Au(111) at this temperature.^9^ However, Br binds to Pd more strongly than it does to Au, with 675 K being the onset temperature required to desorb Br in the atomic form, or as HBr with hydrogen supplied by bulk Pd.^10^ This observation already denotes the presence of Pd‐like sites at the surface. The network retains its porosity until at least 785 K (Figure 2 b and Figure S4), at which temperature we found no evidence for Br atoms in the sCOF pores. Evidence of pores collapsing and complete decomposition of the sCOF was observed after annealing at 885 K (Figure S1). By comparison, Gutzler et al. reported extensive degradation and loss of porosity of the sCOF prepared from TBPB on Cu(111) after thermal annealing at 673 K.^11^ The decomposition on the alloy is likely to be a consequence of two factors. First, annealing induces further palladium enrichment and emergence of active ensembles. Second, the rate of decomposition to surface carbon through dehydrogenation increases at high temperatures.^12^ By contrast, we find that, on Au(111), the framework starts to show signs of decomposition only after annealing at 975 K (Figure S1). These results emphasize the divergence in the robustness of the sCOF on each surface.

**Figure 2:**
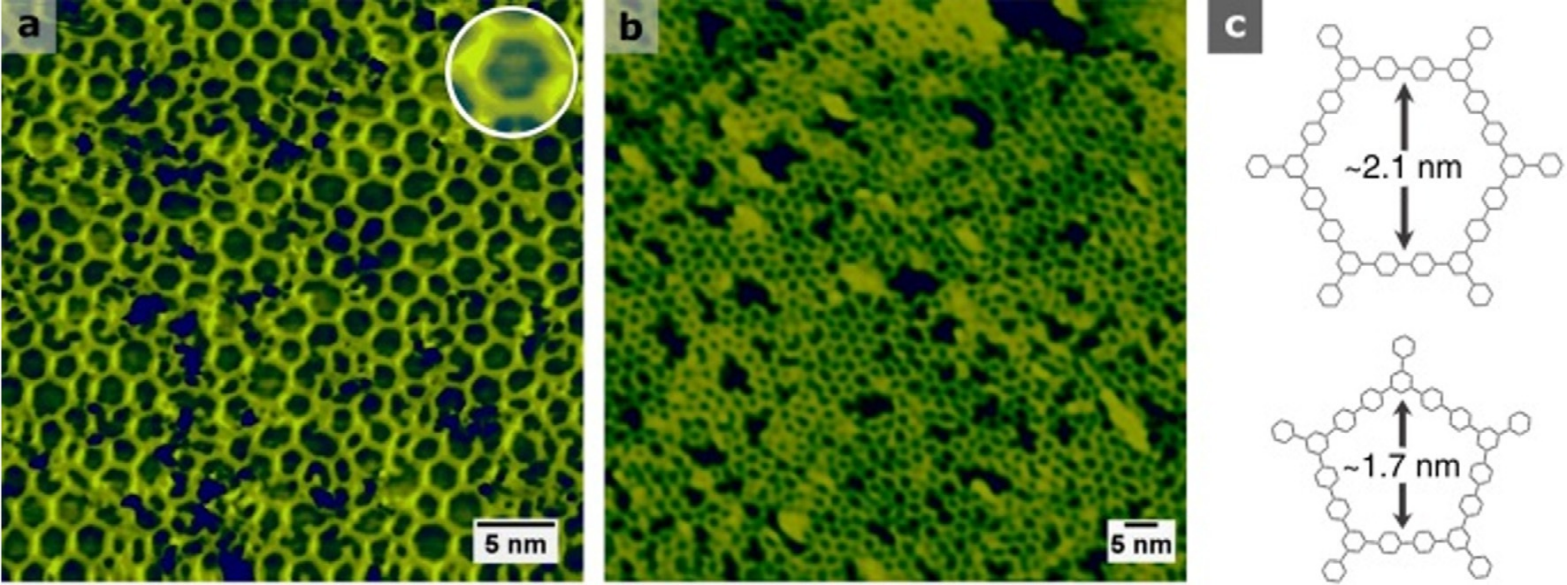
STM images of a) sCOF/Au–Pd(111) post‐annealed for 10 min at 675 K; the inset shows a close‐up of a hexagonal pore populated with Br adatoms. b) After post‐annealing at 785 K (±0.6 V, 100–300 pA). c) Wireframe model of hexagonal and pentagonal pores in the sCOF resulting from aryl C−C coupling (hydrogens are omitted for clarity).

With the purpose of probing the alloying step, the sCOF was exposed to CO (pressure=1×10^−6^ mbar, *t*=10 min) at 300 K, and the surface was analyzed by using reflection absorption infrared spectroscopy (RAIRS) under UHV conditions (Figure 3 a). For the sample annealed at 675 K, negligible CO uptake is evidenced. After annealing at 775 K, two intense peaks emerge at 1905 and 2021 cm^−1^, which are accompanied by two weak features at 2063 and 2140 cm^−1^. Accordingly, we assign the 1905 cm^−1^ band to a *υ*(CO) on Pd bridge sites, and the peak at 2021 cm^−1^ with the shoulder at 2063 cm^−1^ to *υ*(CO) on atop Pd sites.^13^ The former peak is in very good agreement with the earlier, closely analogous, CO RAIRS measurements on clean Au/Pd(111) surfaces; the latter band appears remarkably redshifted compared to the literature value of 2090 cm^−1^ on the clean alloy. This redshift likely arises as a consequence of back donation into the 2π* orbital of CO by the aromatic rings of the sCOF through the surface.^14^ A featureless spectrum below 1900 cm^−1^ suggests the absence of threefold hollow sites, which should only appear for surface alloys of a composition equivalent to or above Au_30_Pd_70_. At 775 K, the composition of an unmodified AuPd alloy is Au_40_Pd_60_. For this composition, the diluting effect of Au restricts the number of Pd_2_ and Pd_3_ clusters, favoring the atop Pd adsorption sites.13a The fact that we observe CO on Pd bridge sites, which have an intensity in the RAIR spectrum that is comparable to that of CO on atop Pd sites, is indicative of palladium enrichment of the surface with respect to clean Au–Pd(111) at this pre‐annealing temperature.^15^ Interestingly, we observe a weak and broad feature emerging at 2140 cm^−1^, which is too blueshifted to be assigned to CO on pure Pd sites, but resembles Pd‐modified atop Au sites. It is well‐known that CO is not stable on Au sites at 300 K, and complete desorption is expected at 255 K.13a A tentative explanation for this feature could be the entrapment of CO underneath the sCOF. Atoms and small molecules, such as CO, are known to intercalate between graphene and metal surfaces. The intercalation process is facilitated by defects in the film, and has also been observed for a porous SiO_2_ film grown on Ru(0001).^16^ We postulate that the pores and defects in our network could provide the diffusion channels for CO permeation and intercalation at the interface of the sCOF and the alloy. However, this notion requires further investigation.

**Figure 3:**
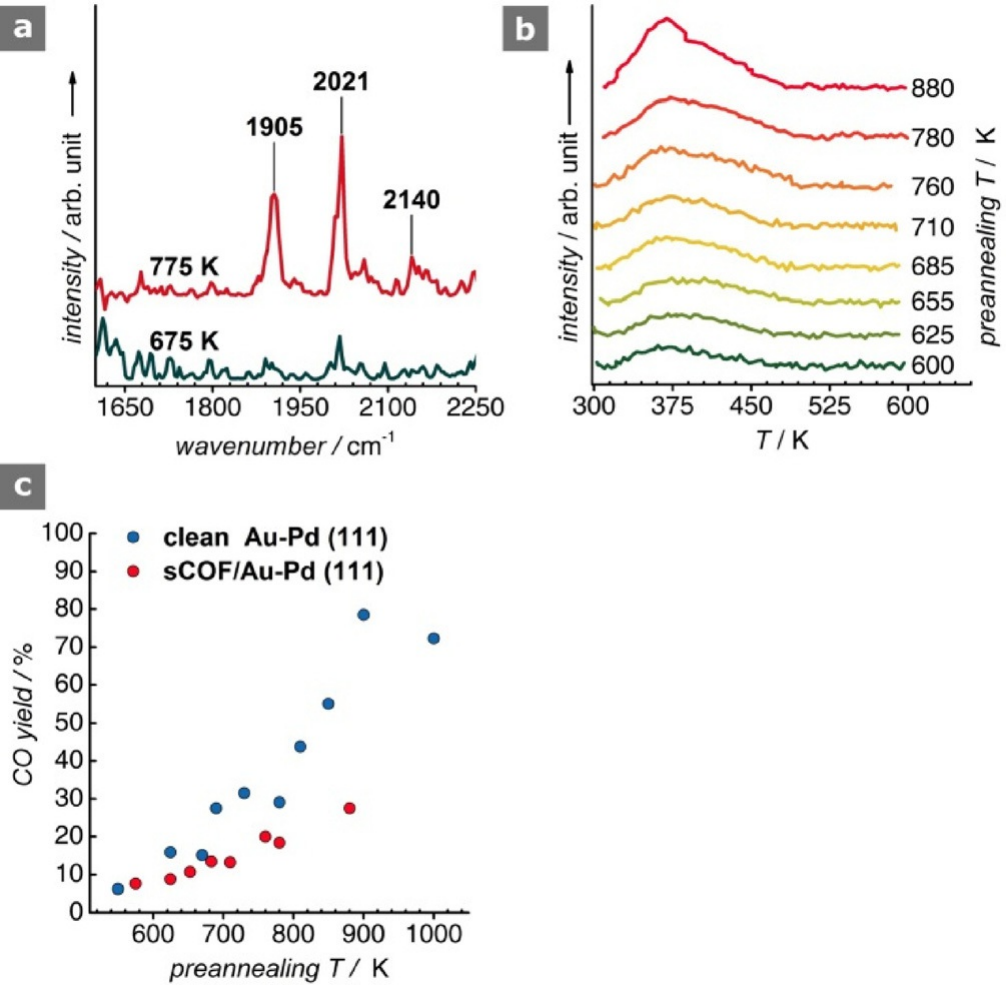
a) RAIR spectra of CO on the 675 and 775 K sCOF/Au‐Pd(111). b) TPD traces for the fragment *m*/*z*=28 (CO) at various pre‐annealing temperatures (*β*=4.1 K s^−1^). c) CO yield derived from the TPD areas and comparison with the clean Au–Pd(111) surface.

Temperature programmed desorption (TPD) spectra show broad traces peaking at *T*_max_≈380 K, which are consistent with CO desorbing from bridge sites (Figure 3 b).13a This correlates well with the band observed at 1905 cm^−1^ in the RAIRS; however, we do not observe a well‐defined peak in the TPD spectra that could indicate CO desorption from atop sites, as opposed to the band detected at 2021 cm^−1^. This inconsistency may find a plausible explanation in the transition of CO from atop to bridge sites upon heating and prior to desorption in the TPD experiments, which has been addressed at length by Kuhn et al.^17^

Marginal amounts of CO desorption for the 600–700 K alloy were detected (ca. 10 %), which increased to around 20 % upon annealing at 760–780 K (Figure 3 c). By comparison, this amount is half that of the yield in the clean Au–Pd(111) system (Figure S5). The lower CO yield is attributed to the blocking of Pd sites by the sCOF, which occupies a significant fraction of the surface, and by the co‐adsorbed Br, which will similarly contribute to this blocking effect.

In conclusion, we have demonstrated that a protocol to fabricate a porous sCOF on catalytically relevant surfaces is feasible under UHV, and the sCOF is sufficiently robust to withstand the temperature required to induce alloying. A marked difference was observed in terms of the thermal stability of the sCOF on the alloy surface compared to a pure Au surface. RAIR and TPD spectra confirmed that the network grown on the Au–Pd(111) surface exhibits accessible Pd sites with palladium enrichment regarding the clean Au–Pd(111) system. Future work will entail the fabrication of functionalized porous networks through Ullmann C−C coupling of pre‐functionalized precursors.

## Experimental Section

Experiments were conducted in two separate stainless‐steel UHV chambers hosting an Ar‐ion sputtering gun and annealing facilities for sample cleaning. TPD data were collected in a UHV chamber equipped with a quadrupole mass spectrometer (SPECTRA, Microvision Plus) in direct line‐of‐sight with the crystal, and a LEED/AES spectrometer (SpectaLEED, Omicron). STM and RAIRS measurements were carried out in a second chamber equipped with a scanning tunneling microscope (VT SPM, Omicron), an infrared spectrometer (Nicolet Magna), and LEED optics. STM images were recorded in constant‐current mode at room temperature by using an electrochemically etched polycrystalline W tip. The voltages stated correspond to the sample bias with respect to the tip. Image processing has been applied to the STM data by using WSxM,^18^ and ImageJ^19^ was used in the pore‐size counting. Images are uncorrected for drift. The RAIR spectra presented is the baseline‐corrected average over 1000 scans at a resolution of 8 cm^−1^. Gold evaporation was achieved by exposing the crystal to a resistively heated Au filament wound around a W wire. TBPB [1,3,5 *tris*(4‐bromophenyl) benzene, Aldrich 97 %] was used as received, and outgassed for 12 h at 340 K prior to being admitted to the chamber. Dosing was achieved by resistively heating a glass microcapillary wrapped in Ta wire containing TBPB at 435 K. The temperature was monitored by using a K‐type thermocouple.

## Supporting information

As a service to our authors and readers, this journal provides supporting information supplied by the authors. Such materials are peer reviewed and may be re‐organized for online delivery, but are not copy‐edited or typeset. Technical support issues arising from supporting information (other than missing files) should be addressed to the authors.

Supplementary
