# Supplementary material for: Fabrication of a High‐Quality, Porous, Surface‐Confined Covalent Organic Framework on a Reactive Metal Surface
Source: Chemphyschem. 2016 Feb 5;17(7):971–5. doi: 10.1002/cphc.201600027 (PMC4991300; doi:10.1002/cphc.201600027)
Supplement: Supplementary file 1 — Supplementary [file CPHC-17-971-s001.pdf]

CHEMPHYSCHEM

Supporting Information

Fabrication of a High-Quality, Porous, Surface-Confined Covalent Organic Framework on a Reactive Metal Surface

Christian R. Larrea and Christopher J. Baddeley*[a]

cphc_201600027_sm_miscellaneous_information.pdf

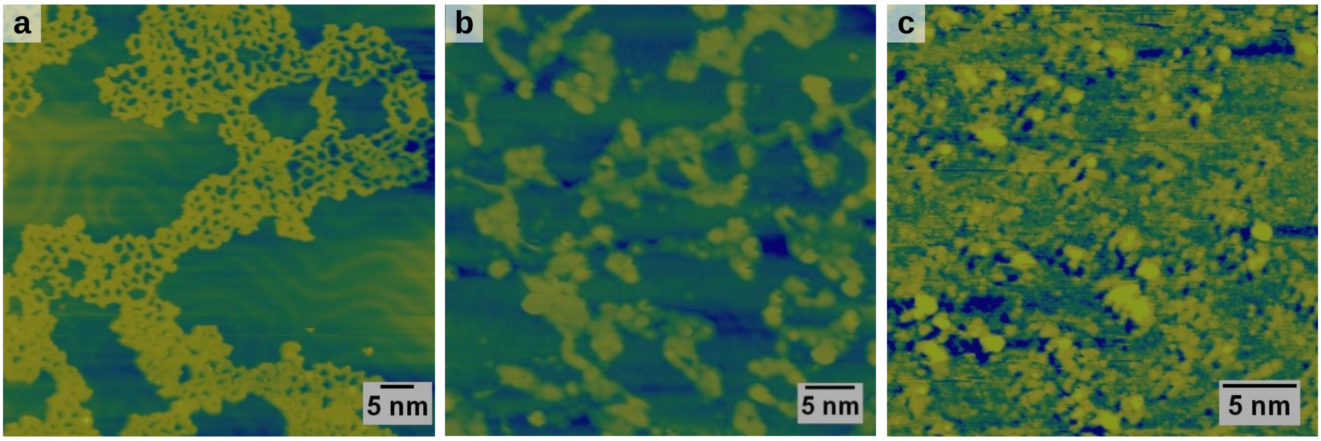

Figure S1. STM images of decomposition on the Au, Pd, and Au-Pd(111) surfaces. (a) sCOF on Au(111) post-annealed to 975 K. (-1.0 V, 100 pA). (b) SCOF/Au-Pd(111) post-annealed to 885 K. (-0.6 V, 300 pA). (c) TBPB deposited on Pd (111) at 475 K (-0.6 V, 300 pA).

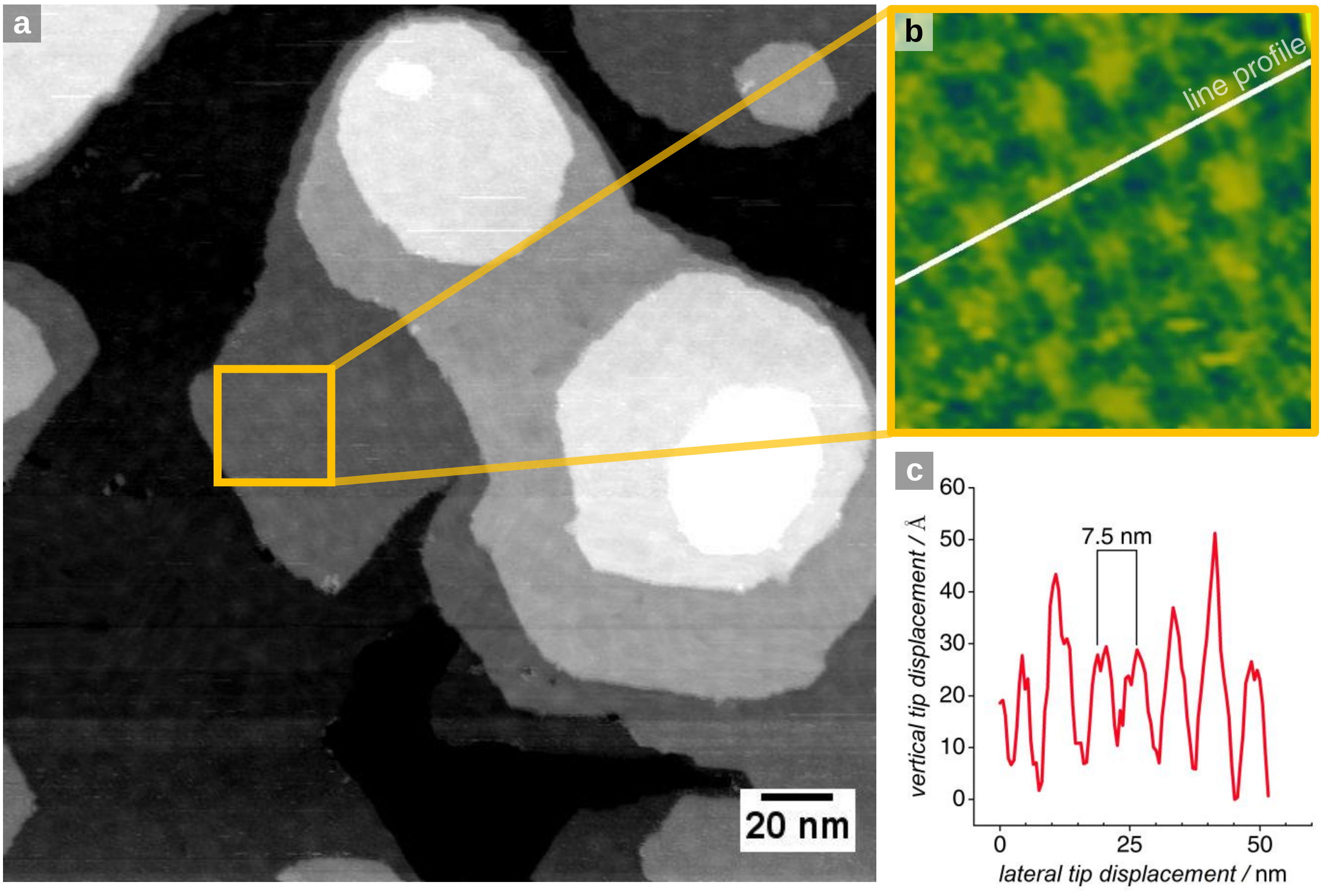

Figure S2. STM image of Au-Pd surface alloy. (a) ~4 monolayer equivalent (MLE) of gold deposited on Pd(111) at 330 K followed by annealing to 575 K for 30 min. (b) Close up of the hexagonal Moiré pattern. (c) line profile highlighting the ~7.5 nm periodicity (-0.6 V, 300 pA).

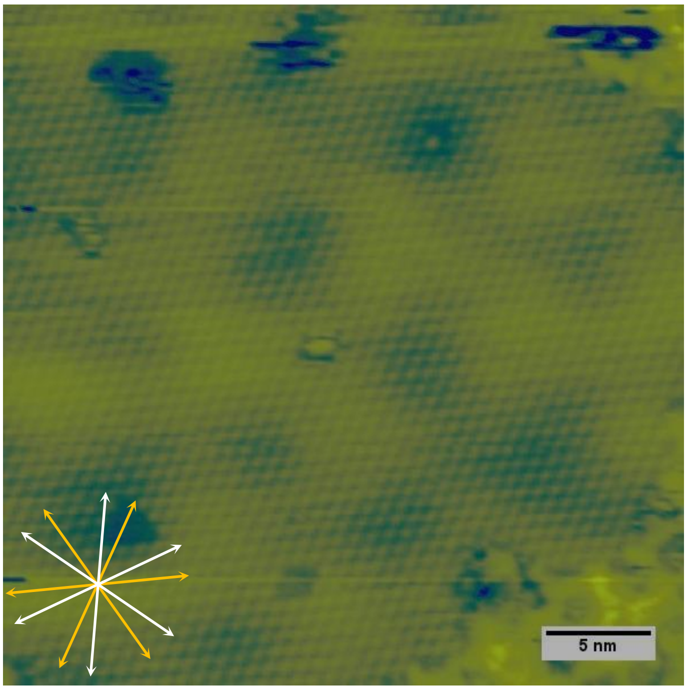

Figure S3. (√7x√7)R 19.1° bromine island. White arrows indicate the directions of the Moiré pattern. Yellow arrows indicate the direction of the close-packed Br overlayer which is rotated ~20° from the Moiré (-0.6 V, 300 pA).

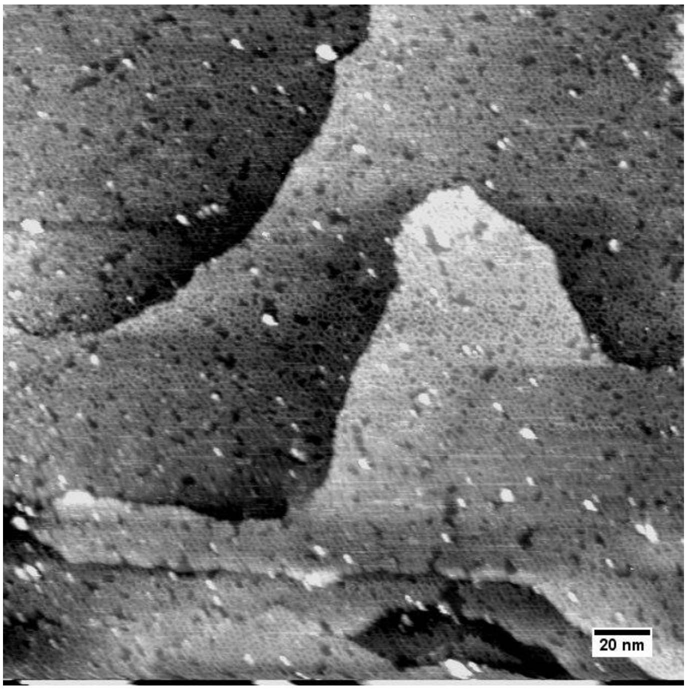

Figure S4. STM image of SCOF/Au-Pd(111) after post-annealing to 785 K. Almost complete coverage of the surface and an intact porous network remains (-0.6 V, 300 pA).

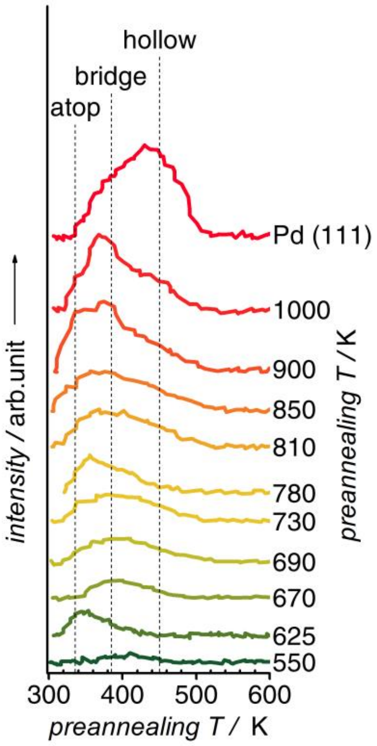

Figure S5. TPD traces for the clean Au-Pd(111) system. Fragment m/z= 28 (CO) at various preannealing temperatures after exposure to 1 x 10^{-6} mbar of CO (β= 5.3 $K{\cdot}s^{-1}$).
